# Activation of **β**-Adrenoceptors by Dobutamine May Induce a Higher Expression of Peroxisome Proliferator-Activated Receptors **δ** (PPAR**δ**) in Neonatal Rat Cardiomyocytes

**DOI:** 10.1100/2012/248320

**Published:** 2012-05-15

**Authors:** Ming-Ting Chou, Shih-Hsiang Lo, Kai-Chun Cheng, Yin-Xiao Li, Li-Jen Chen, Juei-Tang Cheng

**Affiliations:** ^1^Department of Cardiology and Department of Medical Research, Chi-Mei Medical Center, Yong Kang, Tainan 73101, Taiwan; ^2^Department of Cardiology and Internal Medicine, Taipei City Hospital-Zhongxing Branch, Taipei 10341, Taiwan; ^3^Department of Psychosomatic Internal Medicine, Kagoshima University Graduate School of Medical and Dental Sciences, Kagoshima 890-8544, Japan; ^4^Graduate Institute of Basic Medical Science, College of Medicine, National Cheng Kung University, Tainan 70101, Taiwan; ^5^Institute of Medical Sciences, Chang Jung Christian University, Quei-Ren, Tainan 71101, Taiwan

## Abstract

Recent evidence showed the role of peroxisome proliferator-activated receptors (PPARs) in cardiac function. Cardiac contraction induced by various agents is critical in restoring the activity of peroxisome proliferator-activated receptors **δ** (PPAR**δ**) in cardiac myopathy. Because dobutamine is an agent widely used to treat heart failure in emergency setting, this study is aimed to investigate the change of PPAR**δ** in response to dobutamine. Neonatal rat cardiomyocytes were used to examine the effects of dobutamine on PPAR**δ** expression levels and cardiac troponin I (cTnI) phosphorylation via Western blotting analysis. We show that treatment with dobutamine increased PPAR**δ** expression and cTnI phosphorylation in a time- and dose-dependent manner in neonatal rat cardiomyocytes. These increases were blocked by the antagonist of **β**1-adrenoceptors. Also, the action of dobutamine was related to the increase of calcium ions and diminished by chelating intracellular calcium. Additionally, dobutamine-induced action was reduced by the inhibition of downstream messengers involved in this calcium-related pathway. Moreover, deletion of PPAR**δ** using siRNA generated the reduction of cTnI phosphorylation in cardiomyocytes treated with dobutamine. Thus, we concluded that PPAR**δ** is increased by dobutamine in cardiac cells.

## 1. Introduction

Dobutamine is one of the most widely used agents for heart failure in clinic settings [1]. Dobutamine acts through the activation of *β*1-adrenoceptor which is linked to a guanine nucleotide regulatory cascade via heterotrimeric G proteins. This activation results in an increase of adenylyl cyclase activity for the conversion of adenosine triphosphate (ATP) to cyclic AMP (cAMP). An increase in intracellular cAMP concentration causes the release of calcium from sarcoplasmic reticulum. This increased calcium is used by contractile proteins to increase stroke volume [2–6].

Troponin I (TnI) is an inhibitory unit of the troponin complex associated with thin filaments and acts by inhibiting actomyosin interactions in the presence of low levels of intracellular calcium ions (Ca^2+^) during diastole [7, 8]. Modulation of myofilament properties via changes in TnI phosphorylation has profound effects on cardiac contractility and pumping [9]. Phosphorylation of TnI by protein kinase A results in a reduction in myofilament sensitivity to Ca^2+^ and an increase in the cross-bridge cycling rate, resulting in the acceleration of relaxation and an increase in power output, but a reduced economy of contraction [8, 9]. Ca^2+^ is involved in muscle contraction and is an intracellular messenger that activates a wide variety of cellular responses including gene transcription [7–10]. 

Various studies have shown that the activation of calcineurin (Cn) and calcium/calmodulin-dependent protein kinase (CaMK) signaling pathways serves a major role in the regulation of gene expression in cardiac muscles [11, 12]. These same genes have been shown to also be regulated by peroxisome proliferator-activated receptors (PPARs) [13].

PPARs are ligand-activated transcription factors that regulate expression of genes involved in lipid metabolism and inflammation [13]. The three subtypes of PPARs (PPAR*α*, PPAR*γ*, and PPAR*δ*) modulate expression of different genes and exert various bioactivities [13]. Previous studies also showed that metabolic modulators can have beneficial effects in both experimental and clinical heart failure settings [14]. PPAR*δ*-dependent maintenance of cardiac function is crucial for cardiomyocytes [15–17]. The deletion of cardiac PPAR*δ* results in decreased contraction, increased left ventricular end-diastolic pressure, lowered cardiac output, and increased incidences of cardiac failure [15]. Our previous study demonstrated that digoxin enhanced cardiac output by increasing PPAR*δ* expression [18].

Dobutamine is the widely used cardiac agent for patients with heart failure. However, the theory is that the action of dobutamine occurs via the activation of PPAR*δ* remained obscure. In this study, we used the neonatal rat cardiomyocytes to investigate the role of PPAR*δ* in dobutamine-induced action. Moreover, we determined the possible signaling pathways for increase of PPAR*δ* induced by dobutamine.

## 2. Methods

### 2.1. Materials

Dobutamine, atenolol, butoxamine, and cyclosporine A were purchased from Sigma-Aldrich (St Louis, MO, USA). BAPTA-AM and KN93 were purchased from Calbiochem-Novabiochem Corp (La Jolla, CA, USA). The fluorescent probe, Fura2-AM, was obtained from Molecular Probes (Eugene, OR, USA). The Opti-MEM I Reduced Serum Medium, Stealth Select RNAi (siRNA-PPAR*δ*), scramble siRNA (siRNA-control), and Lipofectamine 2000 were purchased from Invitrogen (Carlsbad, CA, USA). Antibodies to PPAR*δ* and actin were purchased from Santa Cruz Biotechnology (Santa Cruz, CA, USA). Antibodies to cardiac TnI and phospho-TnI (Ser 23/24) were purchased from Cell Signaling Technology (Beverly, MA, USA).

### 2.2. Cell Culture

Primary cultures of neonatal rat cardiomyocytes were prepared by the modification of a previously described method [14]. Briefly, under anesthesia with 2% isoflurane, hearts of 1-to-2-day-old Wistar rats were excised, cut into 1-2 mm pieces, and predigested with trypsin to remove red blood cells. The heart tissue was then digested with 0.25% trypsin and 0.05% collagenase. The dissociated cells were placed in uncoated 100 mm dishes and incubated at 37°C in a 5% CO_2_ incubator for at least 1 hour to remove the nonmyocytic cells. This procedure caused fibroblasts to predominantly attach to the dishes while most of the cardiomyocytes remained in suspension. The cardiomyocyte-enriched population was collected and counted. The cells were cultured in Dulbecco/Vogt modified Eagle's minimal essential medium (DMEM) with 1 mmol/L pyruvate, 10% fetal bovine serum (FBS), 100 units/mL penicillin, and 100 units/mL streptomycin. Over 95% of the collected cells were characterized cardiomyocytes on the basis of the sarcomeric myosin content. On the second day, the medium was replaced. After 3 to 4 days in culture, the cells were exposed to hyperglycemic conditions. The high glucose-treated cardiomyocytes were generated by applying 30 mmol/L glucose to the cells for 24 hours [14]. This animal experiment was approved and conducted in accordance with local institutional guidelines for the care and use of laboratory animals in the Chi-Mei Medical Center (number 100052307) and followed the Guide for the Care and Use of Laboratory Animals published by the U.S. National Institutes of Health (NIH Publication number 85-23, revised 1996), as well as the guidelines of the Animal Welfare Act.

### 2.3. Drug Treatment of Cardiomyocytes

Stock solutions of dobutamine were prepared with normal media. Cells were treated with varying concentrations of dobutamine (0.01–10 *μ*mol/L) for 4 hours, washed twice with PBS, and removed by trypsinization. The treated cells were then collected and subjected to a gene expression assay. In addition, pretreatment with various inhibitory agents (*β*1-adenocepotor antagonist (10 *μ*mol/L atenolol) [19, 20], *β*2-adenocepotor antagonist (10 *μ*mol/L butoxamine) [21], calcium chelator (25 mmol/L BAPTA-AM), calcineurin inhibitor (1 *μ*mol/L cyclosporine A) [22], or CaMK inhibitor (1 *μ*mol/L KN-93) [22]) was applied for 30 minutes before the addition of dobutamine. 

### 2.4. Western Blotting Analysis

Protein was extracted from tissue homogenates and cell lysates using ice-cold radio-immunoprecipitation assay (RIPA) buffer supplemented with phosphatase and protease inhibitors (50 mmol/L sodium vanadate, 0.5 mmol/L phenylmethylsulphonyl fluoride, 2 mg/mL aprotinin, and 0.5 mg/mL leupeptin). Protein concentrations were determined with the Bio-Rad protein assay (Bio-Rad Laboratories, Inc., Hercules, CA, USA). Total protein (30 *μ*g) was separated by SDS/polyacrylamide gel electrophoresis (10% acrylamide gel) using the Bio-Rad Mini-Protein II system. Protein was transferred to expanded polyvinylidene difluoride membranes (Pierce, Rockford, IL, USA) with a Bio-Rad Trans-Blot system. After the transfer, the membranes were washed with PBS and blocked for 1 hour at room temperature with 5% (w/v) nonfat dry milk (NFDM) in PBS. Blots were incubated overnight at 4°C with an immunoglobulin-G polyclonal rabbit anti-mouse antibody (Affinity BioReagents, Inc., Golden, CO, USA) diluted 1 : 500 in 5% (w/v) NFDM dissolved in PBS/Tween 20 (0.5% by volume). The blots were also incubated with goat polyclonal antibody (1 : 1000) targeted to actin, which served as an internal control. After the removal of the primary antibody, the blots were extensively washed with PBS/Tween 20. The blots were then incubated for 2 hours at room temperature with the appropriate peroxidase-conjugated secondary antibody diluted in 5% (w/v) NFDM dissolved in PBS/Tween 20. The blots were developed by autoradiography using the ECL-Western blotting system (Amersham International, Buckinghamshire, UK). The immunoblots were quantified with a laser densitometer.

### 2.5. Measurement of Intracellular Calcium Concentration

The changes in intracellular calcium were detected using the fluorescent probe Fura2-AM [23]. Primary cultured cardiomyocytes were placed in a buffered physiological saline solution containing 140 mmol/L NaCl, 5.9 mmol/L KCl, 1.2 mmol/L CaCl_2_, 1.4 mmol/L MgCl_2_, 11.5 mmol/L glucose, 1.8 mmol/L Na_2_HPO_4_, and 10 mmol/L HEPES-Tris. A final concentration of 5 *μ*mol/L Fura-2AM was added to the cells which were incubated for 1 hour in humidified 5% CO_2_ and 95% air at 37°C. The cells were washed and incubated for an additional 30 minutes in PSS. The neonatal rat cardiomyocytes were inserted into a thermostatic (37°C) cuvette containing 2 mL of calcium-free PSS and various doses of dobutamine or inhibitor as previously indicated. The fluorescence was continuously recorded using a fluorescence spectrofluorometer (Hitachi F-2000, Tokyo, Japan). The values of [Ca^2+^]i were calculated from the ratio *R* = F340/F380 by the formula: [Ca^2+^]i = Kd*B* (*R* − *R *_min_)/(*R *_max _− *R*), where Kd is 225 nM, F is the fluorescence, and *B* is the ratio of the fluorescence of the free dye to that of the Ca^2+^-bound dye measured at 380 nm. *R *_max_ and *R*_min⁡_ were determined in separate experiments by using Dobutamine to equilibrate [Ca^2+^]i with ambient [Ca^2+^] (*R *_max_), and the addition of 0.1 mmol/L MnCl_2_ and 1 mmol/L EGTA (*R *_min_). Background autofluorescence was measured in unloaded cells and subtracted from all experimental measurements. 

### 2.6. Small Interfering RNA (siRNA)

Duplexed RNA oligonucleotides for rat PPAR*δ* (Stealth RNAi) were synthesized by Invitrogen using our previous method [24]. The neonatal rat cardiomyocytes were transfected with 40 pmol of PPAR*δ*-specific siRNA (siRNA-PPAR*δ*) or scramble siRNA using Lipofectamine 2000 (Invitrogen) according to the manufacturer's protocols. These cardiomyocytes were subjected to experimental conditions as described above for 48 hours posttransfection. The sequences of the siRNA-PPAR*δ* are UUGCAGAUCCGAUCGCACUUCUCGU (sense strand) and ACGAGAAGUGCGAUCGGAUCUGCAA (antisense strand) as described previously [24].

### 2.7. Statistical Analysis

Statistical analysis was carried out using an ANOVA and the Newman-Keuls post-hoc analysis. Statistical significance was set as *P* < 0.05. The results were expressed as mean ± SEM.

## 3. Results

### 3.1. Increase of PPAR*δ* Expression by Dobutamine in Neonatal Rat Cardiomyocytes

**Figure 1:**
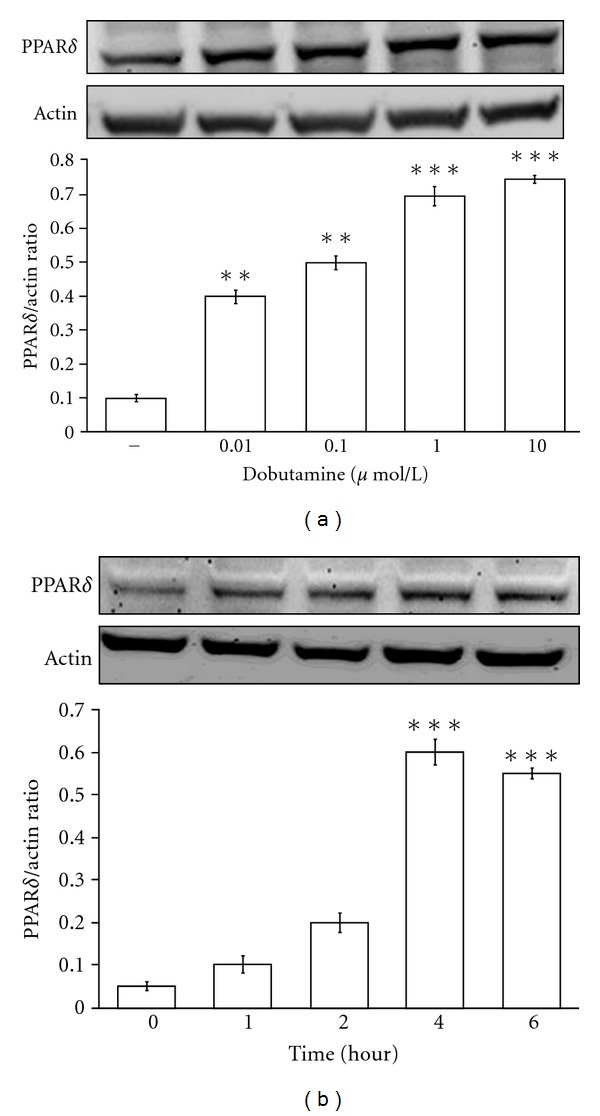
*Effects of dobutamine on PPAR*δ* expression in neonatal rat cardiomyocytes*. The neonatal rat cardiomyocytes were treated with dobutamine at various concentrations for 4 hours (a) or at 1 *μ*mol/L during various time points (b). The treated cells were harvested to determine the protein levels by Western blotting analysis. All values are presented as mean ± SEM (*n* = 6 per group). *P < 0.05 and **P < 0.01 as compared with the vehicle-treated control group.

The neonatal rat cardiomyocytes were treated with dobutamine to identify the changes in PPAR*δ* expression. Treatment with dobutamine at 0.1 *μ*mol/L increased PPAR*δ* protein expression level in a time-dependent manner (Figure 1(b)) and the levels in these cells were increased to maximum at 4 hours later of drug treatment. Dobutamine was then incubated for 4 h at various concentrations ranging from 0.01 to 10 *μ*mol/L. The PPAR*δ* protein expression levels in neonatal rat cardiomyocytes were increased by dobutamine in a concentration-dependent manner (Figure 1(a)).

### 3.2. Effects of Atenolol and Butoxamine on Dobutamine-Induced Actions in Neonatal Rat Cardiomyocytes

**Figure 2:**
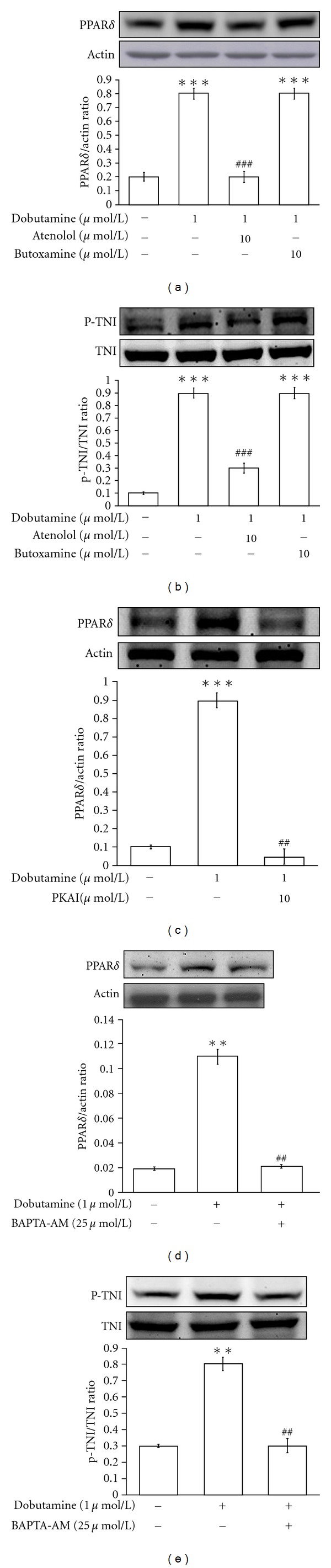
*Effects of receptor antagonists, protein kinase A inhibitor, and calcium chelater on dobutamine-induced actions in neonatal rat cardiomyocytes*. The neonatal rat cardiomyocytes were treated with atenolol, butoxamine, PKAI or BAPTA-AM at indicated concentration for 30 minutes prior to incubation with 1 *μ*mol/L dobutamine. All drugs were dissolved in normal medium. Cells treated with the same volume of normal medium only are indicated as the vehicle-treated control. The treated cells were then harvested to measure PPAR*δ* protein expression (a, c, d) and cTnI phosphorylation (b, e) using Western blotting analysis. All values are expressed as mean ± SEM (*n* = 6 per group). *P < 0.05 as compared with the vehicle-treated control. ^#^ *P* < 0.05 as compared to cells treated with dobutamine only.

To determine the receptor involved in dobutamine-induced the expressions of PPAR*δ* and the phosphorylation of cTnI, we treated the cells with atenolol at a concentration sufficient to block the *β*1-adrenoceptor [19, 20] and butoxamine to block the *β*2-adrenoceptor [21]. The increases in PPAR*δ* expression and cTnI phosphorylation due to dobutamine were inhibited by 10 *μ*mol/L of atenolol (Figures 2(a) and 2(b)); however, butoxamine failed to modify the actions of dobutamine (Figures 2(a) and 2(b)).

### 3.3. Effect of Protein Kinase A Inhibitor on Dobutamine-Induced Actions in Neonatal Rat Cardiomyocytes

The *β*1-adrenoceptor is known to couple to adenylyl cyclase [2–6]. An increase of cyclic AMP causes protein kinase A (PKA) activation which results in higher levels of PPAR*δ* protein expression [25, 26]. Thus, we used the specific inhibitor of protein kinase A (PKAI) to verify the roles of PKA in changes of PPAR*δ* expressions. The dobutamine-induced increases of PPAR*δ* expression were reduced by treatment with 10 *μ*mol/L of PKAI (Figure 2(c)).

### 3.4. Effects of Intracellular Calcium Levels on Dobutamine-Induced Actions in Neonatal Rat Cardiomyocytes

The fluorescent probe, Fura2-AM is used to detect the intracellular calcium concentration in neonatal rat cardiomyocytes. Dobutamine increased the intracellular calcium levels from 155.4 + 11.4 nmol/L to 484.7 + 22.4 nmol/L (*n* = 8) at 0.1 *μ*mol/L and increased in a concentration-dependent manner. Pretreatment of the cells with the calcium chelator BAPTA-AM (BAPTA) at an effective concentration (25 *μ*mol/L) [27] reduced the actions of dobutamine on the increases of PPAR*δ* expression and cTnI phosphorylation (Figures 2(d) and 2(e)).

### 3.5. Calcium-Related Pathway as the Possible Signal for Dobutamine-Induced Actions in Neonatal Rat Cardiomyocytes

**Figure 3:**
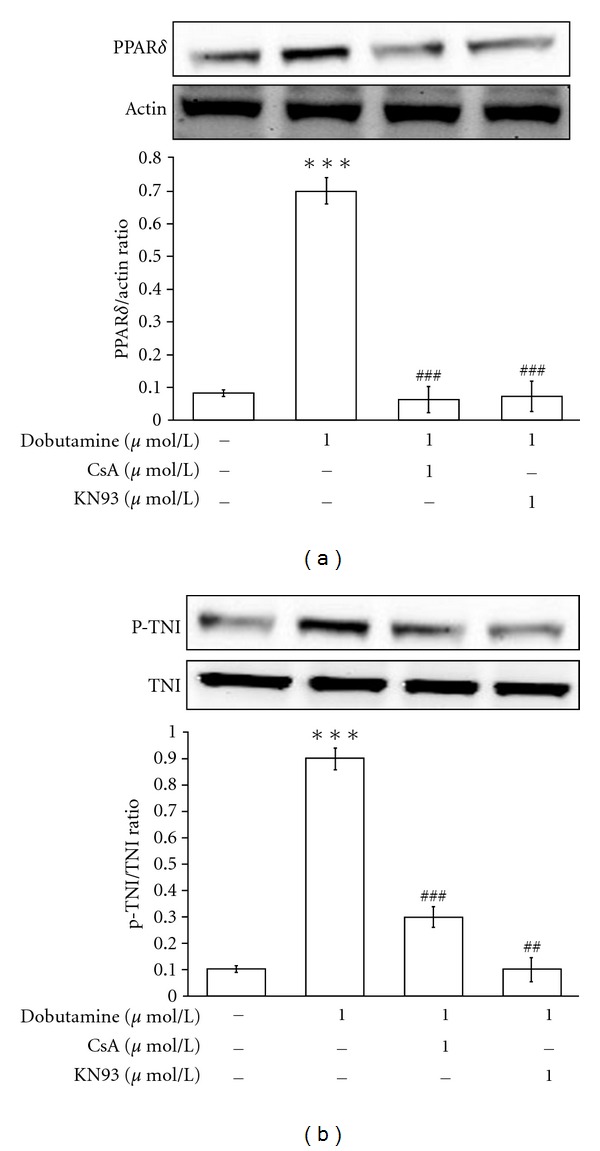
*Effects of calcium-mediated signaling inhibitors on dobutamine-induced actions in neonatal rat cardiomyocytes*. The neonatal rat cardiomyocytes were treated with 1 *μ*mol/L cyclosporine A (CsA, calcineurin inhibitor) or 1 *μ*mol/L KN93 (CaMK inhibitor) at 30 minutes prior to incubation with 1 *μ*mol/L dobutamine for 4 hours. The cells were then harvested to measure PPAR*δ* protein expression (a) and cTnI phosphorylation (b) using Western blotting analysis. All values are expressed as mean ± SEM (*n* = 6 per group). **P < 0.01 as compared with control cells. ^##^ *P* < 0.01 as compared to the control cells incubated with dobutamine only.

Cyclosporine A (CsA, a calcineurin inhibitor) and KN93 (a calcium/calmodulin kinase inhibitor) were used to investigate whether dobutamine-induced PPAR*δ* expression is mediated through the activation of calcium-related pathway [22]. The dobutamine-induced increases in PPAR*δ* expression and cTnI phosphorylation were markedly inhibited by either 1 *μ*mol/L of CsA or 1 *μ*mol/L of KN93 (Figures 3(a) and 3(b)).

### 3.6. Effects of siRNA-PPAR*δ* on Dobutamine-Induced Actions in Neonatal Rat Cardiomyocytes

**Figure 4:**
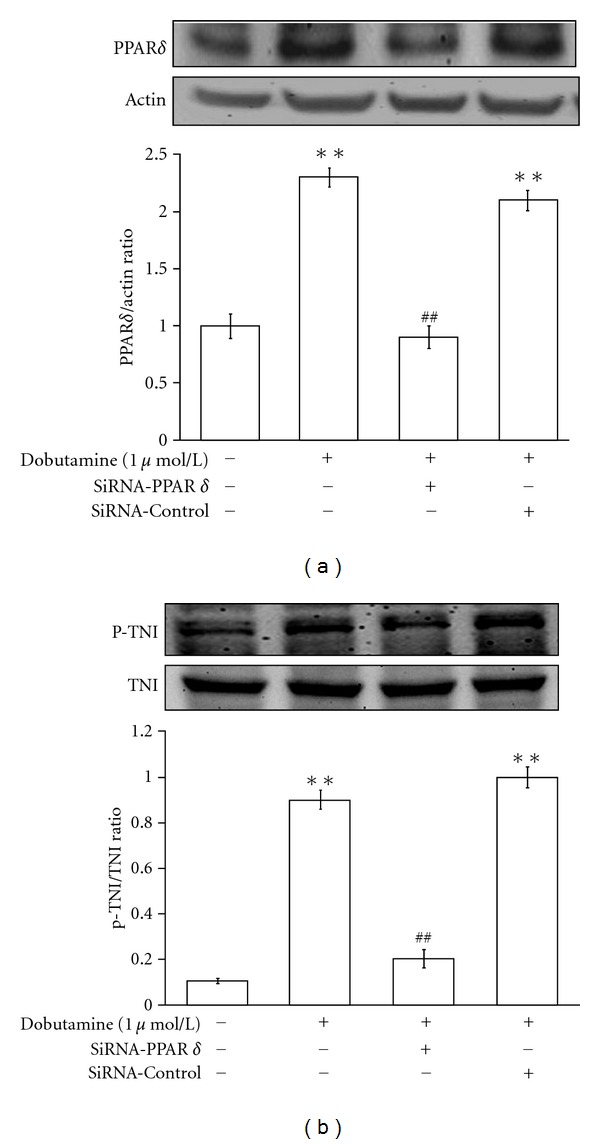
*Effects of PPAR*δ*-targeted siRNA on dobutamine-induced actions in neonatal rat cardiomyocytes*. The neonatal rat cardiomyocytes transfected with siRNA-targeted to PPAR*δ* or siRNA-scramble (siRNA-control) were incubated with 1 *μ*mol/L dobutamine. All cells were then harvested to measure PPAR*δ* expression (a) or cTnI phosphorylation (b) using Western blotting analysis. All values are expressed as mean ± SEM (*n* = 6 per group). *P < 0.05 as compared with the vehicle-treated control and ^#^ *P* < 0.05 as compared with the dobutamine-treated cells transfected with the scramble siRNA (siRNA-control).

 The neonatal rat cardiomyocytes were transfected with either siRNA targeted to PPAR*δ* or a scramble control for 48 hours, as described previously [24]. After treatment with dobutamine, PPAR*δ* expression was markedly reduced in the cardiomyocytes that were transfected with the siRNA targeted to PPAR*δ* (Figure 4(a)). The cells transfected the siRNA-scramble did not affect dobutamine-induced increase of PPAR*δ* expression (Figure 4(a)). Moreover, dobutamine-induced TnI phosphorylation was also reduced in cells treated with siRNA-PPAR*δ* but remained unchanged in the cells transfected with siRNA-scramble (Figure 4(b)).

## 4. Discussion

In the present study, treatment with dobutamine in neonatal rat cardiomyocytes caused a concentration-dependent increase in both PPAR*δ* protein expression and cTnI phosphorylation. These increases induced by dobutamine were blocked by pretreatment with atenolol, PKAI, KN93, CsA, or BAPTA. Moreover, the dobutamine-induced increases in PPAR*δ* protein expression and cTnI phosphorylation were markedly reduced in neonatal rat cardiomyocytes that were transfected with siRNA targeted to PPAR*δ*. Thus, the mediation of PPAR*δ* in dobutamine-induced cardiac action can be considered. It has been reported that PPAR**δ** is involved in excitation-transcription coupling [28] and that calcineurin-mediated skeletal muscle reprogramming induces the expression of several transcriptional regulators, including PPAR**δ** [29]. Taken together, we suggest that the increase in PPAR**δ** expression by dobutamine is mainly induced by an activation of the *β*1-adrenoceptor, which results in an increase of intracellular cAMP and calcium. This leads to an increase in heart contractility.

Regulation of PPAR*δ* expression in cardiac muscles through the intracellular Ca^2+^ signaling pathway has been established [22, 30, 31]. We show that treatment with BAPTA suppressed dobutamine-induced PPAR*δ* protein expression. We also show that the inhibition of PKA reduced dobutamine-induced expression of PPAR*δ*. This result is consistent with the finding that the activation of PKA induces intracellular calcium release [32]. Thus, dobutamine exerts its effects on PPAR*δ* expression in a calcium-dependent manner via the activation of PKA in cardiac cells.

It has been demonstrated that cTnI phosphorylation most likely occurs due to an enhanced off rate during Ca^2+^ exchange with the cardiac troponin calcium binding site, leading to an acceleration of relaxation and an increase in cardiac output [31, 33–36]. Similarly, we found that cTnI phosphorylation is elevated in neonatal rat cardiomyocytes after treatment with dobutamine. We also observed that pretreatment with calcium chelater (BAPTA) decreased the levels of cTnI phosphorylation in dobutamine-treated cardiomyocytes. Therefore, we suggest that the increase in intracellular calcium is responsible for the increase of cTnI phosphorylation by dobutamine. This explanation is consistent with previously published reports [31, 33–36].

The role of PPAR*δ* in the phosphorylation of cTnI in cardiomyocytes remains unclear. Thus, we applied PPAR*δ*-targeted siRNA to better characterize this possible relationship. In this study, a 48 hours transfection of siRNA-PPAR*δ* in cardiomyocytes suppressed dobutamine-induced cTnI phosphorylation. Thus, it is suggested that PPAR*δ* is involved in dobutamine-induced cTnI phosphorylation in cardiomyocytes.

In conclusion, we demonstrated that treatment with dobutamine in neonatal rat cardiomyocytes can increase PPAR*δ* expression by an activation of the *β*1-adrenoceptor through cAMP to activate PKA and increase intracellular calcium levels. This increase may induce calmodulin and calcineurin activation which could result in higher PPAR*δ* protein expression. Taken together, increases in PPAR*δ* protein expression and cTnI phosphorylation are responsible for dobutamine-induced cardiac action, suggesting a new mechanism for dobutamine-induced cardiac contraction.

## Abbreviations

PPAR*δ*: Peroxisome proliferator-activated receptors *δ*
cTnI: Cardiac troponin I
ATP: Adenosine triphosphate
cAMP: Cyclic adenosine monophosphate
TnI: Troponin I
Cn: Calcineurin
CaMK: Calcium/calmodulin-dependent protein kinase
PPARs: Peroxisome proliferator-activated receptors
DMEM: Dulbecco/Vogt modified Eagle's minimal essential medium
FBS: Foetal bovine serum
RIPA: Radio-immuno-precipitation assay
BAPTA: BAPTA-AM
IBMX: 3-isobutyl-1-methylxanthine
PKA: Protein kinase A
PKAI: Inhibitor of protein kinase A
CsA: Cyclosporine A.
